# The Early Clinical Features of Dengue in Adults: Challenges for Early Clinical Diagnosis

**DOI:** 10.1371/journal.pntd.0001191

**Published:** 2011-05-31

**Authors:** Jenny G. H. Low, Adrian Ong, Li Kiang Tan, Shera Chaterji, Angelia Chow, Wen Yan Lim, Koon Wui Lee, Robert Chua, Choon Rong Chua, Sharon W. S. Tan, Yin Bun Cheung, Martin L. Hibberd, Subhash G. Vasudevan, Lee-Ching Ng, Yee Sin Leo, Eng Eong Ooi

**Affiliations:** 1 Communicable Diseases Centre, Tan Tock Seng Hospital, Singapore, Singapore; 2 Environmental Health Institute, National Environment Agency, Singapore, Singapore; 3 Duke-NUS Graduate Medical School, Singapore, Singapore; 4 DSO National Laboratories, Singapore, Singapore; 5 Singapore Clinical Research Institute, Singapore, Singapore; 6 Genome Institute of Singapore, Singapore, Singapore; University of California, Berkeley, United States of America

## Abstract

**Background:**

The emergence of dengue throughout the tropical world is affecting an increasing proportion of adult cases. The clinical features of dengue in different age groups have not been well examined, especially in the context of early clinical diagnosis.

**Methodology/Principal Findings:**

We structured a prospective study of adults (≥18 years of age) presenting with acute febrile illness within 72 hours from illness onset upon informed consent. Patients were followed up over a 3–4 week period to determine the clinical outcome. A total of 2,129 adults were enrolled in the study, of which 250 (11.7%) had dengue. Differences in the rates of dengue-associated symptoms resulted in high sensitivities when the WHO 1997 or 2009 classification schemes for probable dengue fever were applied to the cohort. However, when the cases were stratified into age groups, fewer older adults reported symptoms such as myalgia, arthralgia, retro-orbital pain and mucosal bleeding, resulting in reduced sensitivity of the WHO classification schemes. On the other hand, the risks of severe dengue and hospitalization were not diminshed in older adults, indicating that this group of patients can benefit from early diagnosis, especially when an antiviral drug becomes available. Our data also suggests that older adults who present with fever and leukopenia should be tested for dengue, even in the absence of other symptoms.

**Conclusion:**

Early clinical diagnosis based on previously defined symptoms that are associated with dengue, even when used in the schematics of both the WHO 1997 and 2009 classifications, is difficult in older adults.

## Author Summary

Dengue infection in adults has become increasingly common throughout the world. As most of the clinical features of dengue have been described in children, we undertook a prospective study to determine the early symptoms and signs of dengue in adults. We show here that, overall, dengue cases presented with high rates of symptoms listed in the WHO 1997 or 2009 classification schemes for probable dengue fever thus resulting in high sensitivities of these schemes when applied for early diagnosis. However, symptoms such as myalgia, arthralgia, retro-orbital pain and mucosal bleeding were less frequently reported in older adults. This trend resulted in reduced sensitivity of the WHO classification schemes in older adults even though they showed increased risks of hospitalization and severe dengue. Instead, we suggest that older adults who present with fever and leukopenia should be tested for dengue, even in the absence of other symptoms. This could be useful for early clinical diagnosis in older adults so that they can be monitored and treated for severe dengue, which is especially important when an antiviral drug becomes available.

## Introduction

The mosquito-borne dengue virus (DENV) has emerged in the latter half of the 20^th^ century to become an important cause of morbidity and mortality. Over half of the world's population live at risk of infection annually [1]. Infection with any of the four antigenically distinct virus serotypes results in a wide range of clinical manifestation, from mild undifferentiated febrile illness to classical dengue fever (DF) to the life-threatening dengue hemorrhagic fever (DHF) and dengue shock syndrome (DSS) [2]; the latter two syndromes are characterized by plasma leakage resulting from alteration in microvascular permeability [3], [4], [5]. Supportive fluid therapy is effective in preventing the onset of shock from excessive plasma leakage but relies on early diagnosis of dengue and monitoring for the clinical signs of plasma leakage [5]. Furthermore, early diagnosis would have an increasingly important role with the development of antiviral therapies, because the effectiveness of antivirals is likely to be high only if initiated early after illness onset [6], during the short viremic phase [7], [8].

Differentiating dengue from other causes of febrile illness clinically is difficult to achieve reliably during the early phase of illness [9]. In most dengue endemic countries, access to diagnostic laboratories is limited and dengue diagnosis may rely solely on clinical recognition. Moreover, even where diagnostic laboratory services are available, virological tests are requested only upon a clinical suspicion of dengue, based on the presenting symptoms and signs. The World Health Organization (WHO) developed a set of guidelines (WHO 1997) [10], which was recently revised (WHO 2009) [11], to aid diagnosis and disease classification for case management, but how these schemes perform in the context of early clinical diagnosis needs further evaluation. Furthermore, dengue infection in adults is showing an increasing trend globally, both among travellers [12], [13] as well as those residing in endemic regions [14], [15], [16], [17], [18], [19], [20]. The collective clinical experience of dengue in adults is limited compared to that in children [5], [21], [22], [23], [24], upon which the criteria for dengue diagnosis in the WHO classification schemes have been developed. Adults appear to be at lower risk of DHF compared to children [25], but complications such as bleeding and severe organ impairment are more common [26], [27], [28], [29]. How increasing age affects the clinical presentation of dengue infections and hence early clinical dengue diagnosis, is unknown.

We undertook a multi-centre longitudinal study of adult dengue infection to characterize the early phase of dengue illness. We report here the observations obtained from 2,129 patients enrolled over a five-year period. Our findings indicate that the symptoms associated with dengue are less frequently reported in older adults, making early clinical diagnosis more difficult with increasing age of the cases.

## Materials and Methods

### Study design and patients

The study protocol was approved by the National Healthcare Group Domain Specific Review Board (DSRB B/05/013), as well as the Institutional Review Boards of the National University of Singapore and DSO National Laboratories. Enrolment of study participants was conditional on appropriate written informed consent administered by a study research nurse. All biological material collected were de-identified after completion of demographic and clinical data collection.

### Screening and recruitment

The protocol for patient recruitment was described previously [30]. Adult patients (age ≥18 years) presenting with acute onset fever (a presenting temperature of ≥37.5°C or a history of fever ≥37.5°C for less than 72 hours) at selected public primary healthcare clinics were eligible for study inclusion. Upon consent, demographic, clinical and epidemiological information were collected on a standardized data entry form on three occasions: 1–3 days post fever onset (Visit 1), 4–7 days post fever onset (Visit 2) and 3–4 weeks post fever onset (Visit 3) during convalescence [30]. Venous blood was collected at every visit. Serum was stored at −80°C until use. From January 2008, a nasal swab was also collected, which was immediately placed in viral transport medium, and tested for respiratory pathogens in the laboratory.

Recruited patients who were hospitalized for further management within the defined study period were followed up in the hospital or post-discharge. These patients received medical treatment at the discretion of their attending physicians. Hospitalization information and investigation data were extracted from electronic hospital records or discharge summaries using a pre-defined protocol.

### Hospital admission criteria

The decision to hospitalize a patient was left to the discretion of the treating physician. However, national guidelines on dengue management are available and are adopted by the healthcare institutions in Singapore [31]. Hospitalization criteria in these guidelines include: significant bleeding, fall in blood pressure, dehydration and postural hypotension, rise in hematocrit of 20% or greater compared to the baseline, platelet count <80,000 cells/mm^3^, severe vomiting or diarrhea, severe abdominal pain, and elderly patients with co-morbidities who are unwell.

### WHO dengue classification schemes

The WHO 1997 [10] and WHO 2009 [11] classification schemes were applied to the clinical data obtained at Visit 1. WHO 1997 [10] classifies acute febrile illness as probable DF based on headache, retro-orbital pain, myalgia, arthralgia, rash, hemorrhagic manifestations and leukopenia. In contrast, WHO 2009 [11] utilizes two or more clinical manifestations for a probable DF classification, which are, nausea/vomiting, rash, aches and pains, tourniquet test positive, leukopenia and any warning signs. For this analysis, leukopenia was defined as a white blood cell count (WBC) of below 4,500 cells/µL and joint pain/muscle pain was included under aches and pains. The tourniquet test was not carried out in the study and hence was not included in the analysis. Three of the seven warning signs [11], namely clinical fluid accumulation, liver enlargement >2cm and increase in haematocrit concurrent with rapid decrease in platelet count were also not included for analysis as these parameters are not routinely monitored by primary healthcare clinicians in patients presenting with acute febrile illness. Likewise, persistent vomiting could not be assessed solely on a single visit. Drowsiness was counted under lethargy/restlessness. Hence, the warning signs used in this analysis were abdominal pain, mucosal bleed, and drowsiness. The WHO classification schemes and the factors included in this study are summarized in Table 1.

**Table 1:** The WHO 1997 and 2009 classification schemes and the parameters used in this study.

WHO 1997		WHO 2009	
Defined in [10]	Used in this study	Defined in [11]	Used in this study
Fever and 2 or more of the following:	Fever and 2 or more of the following:	Fever and 2 or more of the following:	Fever and 2 or more of the following:
Headache	Headache	Anorexia	Loss of appetite
Retro-orbital pain	Retro-orbital pain	Nausea	Nausea
Mylagia	Mylagia	Rash	Rash
Arthralgia	Arthralgia	Aches and pains	Myalgia
Rash	Rash		Arthalgia
Bleeding	Bleeding	Leukopenia	Leukopenia
Leukopenia	Leukopenia	Positive tourniquet test	
			
		Warning signs:	Warning signs:
		Abdominal pain or tenderness	Abdominal pain
		Mucosal bleed	Mucosal bleed
		Lethargy or restlessness	Drowsiness
		Persistent vomiting	*Other warning signs were not used as they require repeated monitoring or are late clinical signs. See text for explanation*.
		Clinical fluid accumulation	
		Liver enlargement >2 cm	
		Laboratory: increase in hematocrit concurrent with a rapid decrease in platelet count	

### Hematology

A full blood count was performed on anticoagulated blood collected at all time points using a bench-top, FDA-approved hematocytometer (iPoch-100, Sysmex, Japan).

### Serology

IgM and IgG antibodies against DENV were detected using commercially available ELISAs (Panbio, Brisbane, Australia) according to manufacturer's instructions.

### Reverse-transcription polymerase chain reaction (RT-PCR)

RT-PCR was performed to detect DENV RNA and determine the serotype of the DENV as previously described [32]. Results were analyzed with the LightCycler software version 3.5. Reactions with high crossover point (Cp) or ambiguous melting curve results were further analyzed by 2% agarose gel electrophoresis, to confirm the presence of the correctly sized amplicon.

RT-PCR was also carried out to test for influenza A and B viruses in the nasal swabs. Briefly, viral RNA was extracted from the viral transport medium using QIAamp Viral RNA mini kit (Qiagen, Hilden, Germany) according to the manufacturer's protocol. Viral RNA was reversed transcribed using random hexamer primers with Superscript III (Invitrogen) according to the manufacturer's protocol. Influenza A and B viruses were detected using previously described methods [33], [34].

### Statistical analysis

Statistical analysis was performed using GraphPad Prism v5.0 d. For continuous variables, the Mann Whitney U test was applied to determine statistical significance. If more than two groups of continuous variables were analysed, the Kruskal-Wallis test was used. Fisher's exact test and the chi-square test with Yates' continuity correction were used in comparisons of sensitivity and specificity as well as rates of presenting symptoms, pre-existing co-morbidities, hospitalization and severe dengue. All analyses were two-tailed. A *P* value of less than 0.05 was considered statistically significant.

## Results

A total of 2,129 patients were enrolled from April 2005 to August 2010. Of these, 246 were RT-PCR positive for dengue while an additional 4 patients tested positive for dengue IgM antibody in the convalescent but not acute sera. All 250 (11.7%) patients were considered as having acute dengue infection. The remaining 1,879 were both RT-PCR and serologically negative for dengue and were thus classified as having other febrile illnesses (OFI) in our analyses. Since January 2008, nasal swabs have been collected and tested for respiratory viruses to further differentiate those with OFI. A total of 228 influenza A and B infections were identified and included as a sub-analysis to identify features that are more specific to dengue infections. Baseline demographic data and virological information of the study population are shown in Table 2.

**Table 2:** Demographics of the dengue, OFI and influenza patients enrolled into the study.

	Dengue N = 250	OFI N = 1879	P value	Influenza N = 228	P value
Median age (25^th^ and 75^th^ percentiles)	39.0 (28.0–49.3)	33.0 (24.0–47.0)	<0.0001*	28.0 (22.0–45.0)	<0.0001*
Males (%)	152 (60.8)	1168 (62.2%)	NS	143 (62.7)	NS
Ethnicity					
Chinese (%)	178 (71.2)	1108 (59.0)	0.0004	111 (48.7)	<0.0001
Malays (%)	18 (7.2)	302 (16.1)		37 (16.2)	
Indians (%)	34 (13.6)	288 (15.3)		39 (17.1)	
Others (%)	20 (8.0)	181 (9.6)		41 (18.0)	
Pre-existing co-morbidity (%)	42 (16.8)	268 (14.3)	NS	24 (10.5)	NS
Asthma (%)	2 (0.8)∧	6 (0.3)	NS	0	NS
Diabetes mellitus (%)	9 (3.6)∧	81 (4.3)∧	NS	7 (3.1)∧	NS
Hypertension (%)	24 (9.6)∧	191 (10.2)∧	NS	21 (9.2%)	NS
Ischemic heart disease (%)	1 (0.4)∧	26 (1.3)∧	NS	6 (2.6)	NS
Others (%)	12 (4.8)	35 (1.9)	NS	1 (0.4)	NS

All P values shown are analysed in comparison to dengue. Chi-square test with Yates' continuity correction was used to determine statistical significance for all parameters except mean age.

*Mann Whitney U test was used to determine P value.

∧Some patients in these categories have more than one pre-existing co-morbidities.

### Comparison of dengue, OFI and influenza

The clinical features of dengue, OFI and influenza at Visit 1 are shown in Table 3. Arthralgia, loss of appetite, nausea, vomiting, altered taste sensation, rashes and skin sensitivity were more frequently reported in patients with dengue compared to OFI and influenza (Table 3). Presenting aural temperature was higher in patients with dengue compared to OFI but not influenza. The mean platelet, WBC, lymphocyte and neutrophil counts were significantly lower in dengue compared to OFI or influenza (Table 4).

**Table 3:** The early clinical features and outcomes of dengue, OFI and influenza.

	Dengue N = 250	OFI N = 1879	RR (95% C.I.)	P value	Influenza N = 228	RR (95% C.I.)	P value
Drowsiness (%)	148 (59.2)	971 (51.7)	1.1 (1.0–1.3)	0.03	126 (55.3)	1.1 (0.9–1.3)	NS
Headache (%)	200 (80.0)	1290 (68.7)	1.2 (1.1–1.3)	0.0003	176 (77.2)	1.0 (0.9–1.1)	NS
Myalgia (%)	173 (69.2)	1197 (63.7)	1.1 (1.0–1.2)	NS	149 (65.4)	1.1 (0.9–1.2)	NS
Arthralgia (%)	152 (60.8)	800 (42.6)	1.4 (1.3–1.6)	<0.0001	82 (36.0)	1.7 (1.4–2.0)	<0.0001
Loss of appetite (%)	203 (81.2)	1126 (59.9)	1.4 (1.3–1.5)	<0.0001	146 (64.0)	1.3 (1.1–1.4)	<0.0001
Abdominal pain (%)	29 (11.6)	276 (14.7)	0.8 (0.5–1.1)	NS	34 (14.9)	0.8 (0.5–1.2)	NS
Diarrhea (%)	37 (14.8)	175 (9.3)	1.6 (1.1–2.2)	0.0091	12 (5.3)	2.8 (1.5–5.3)	0.0008
Nausea (%)	125 (50.0)	543 (28.9)	1.7 (1.5–2.0)	<0.0001	75 (32.9)	1.5 (1.2–1.9)	0.0002
Vomiting (%)	41 (16.4)	158 (8.4)	2.0 (1.4–2.7)	<0.0001	17 (7.5)	2.2 (1.3–3.8)	0.0031
Altered taste (%)	203 (81.2)	1070 (56.9)	1.4 (1.3–1.5)	<0.0001	143 (62.7)	1.3 (1.2–1.5)	<0.0001
Retro-orbital pain (%)	65 (26.0)	298 (15.9)	1.6 (1.3–2.1)	<0.0001	50 (21.9)	1.2 (0.9–1.6)	NS
Rashes (%)	24 (9.6)	67 (3.6)	2.7 (1.7–4.2)	<0.0001	0	Infinity	<0.0001
Bleeding (%)	13 (5.2)	33 (1.76)	3.0 (1.6–5.6)	0.0010	6 (2.6)	2.0 (0.8–5.1)	NS
Aural temp °C∧	38.3 (37.7–39.0)	38.0 (37.3–38.5)	-	<0.000*	38.4 (38.0–38.9)	-	NS*
Total days of illness∧	10 (7–14)	5 (3–7)	-	<0.0001*	6 (4–7)	-	<0.0001*
Hospitalized (%)	116 (46.4)	26 (1.4)	33.5 (22.4–50.2)	<0.0001	2 (0.9)	52.9 (13.2–211.6)	<0.0001

∧Shown are median values with the 25^th^ and 75^th^ percentiles in parentheses.

Chi-square test with Yates' continuity correction was used except where indicated. *Mann Whitney U test.

*Mann Whitney U test.

**Table 4:** The early laboratory parameters of dengue, OFI and influenza infection.

	Dengue N = 250	OFI N = 1879	RR (95% C.I.)	P value	Influenza N = 228	RR (95% C.I.)	P value
Hb g/dL∧	14.9 (13.3–16.1)	14.6 (13.1–15.9)	-	NS*	14.9 (13.3–16.3)	-	NS*
HCT%∧	44.0 (39.5–47.7)	44.0 (39.9–47.5)	-	NS*	45.1 (41.1–49.0)	-	0.0160*
Platelet count×103/µL∧	163.5 (119.0–212.0)	239.0 (191.0–291.0)	-	<0.0001*	219.0 (163.0–261.0)	-	<0.0001*
WBC×103/µL∧	3.8 (2.7–5.1)	7.3 (5.6–9.8)	-	<0.0001*	6.6 (4.8–8.2)	-	<0.0001*
Lymphocyte count×103/µL∧	0.5 (0.4–0.8)	1.2 (0.8–1.7)	-	<0.0001*	1.0 (0.7–1.3)	-	<0.0001*
Neutrophil count×103/µL∧	3.0 (2.0–4.2)	5.5 (3.9–7.8)	-	<0.0001*	5.2 (3.5–6.7)	-	<0.0001*

∧Shown are median values with the 25^th^ and 75^th^ percentiles in parentheses.

Chi-square test with Yates' continuity correction was used except where indicated.

*Mann Whitney U test.

Hb indicates hemoglobin concentration. HCT indicates hematocrit. WBC indicates white blood cell count.

Dengue patients experienced a longer duration of illness compared to OFI and influenza and a higher proportion of them (46.4%) were hospitalized (Table 3). The factors identified at Visit 1 that were associated with hospitalization are shown in Table 5. Hospitalized dengue cases were significantly older than those that received only ambulatory care. The platelet, WBC, lymphocyte and neutrophil counts were significantly lower in hospitalized compared to ambulatory patients. In addition, a higher rate of secondary infection, as defined by a positive DENV IgG finding on the blood sample taken at Visit 1, and a lower crossover point of the real-time RT-PCR, which is indicative of higher viremia levels, were also observed in the hospitalized patients (Table 5).

**Table 5:** Laboratory parameters in dengue patients who received hospitalized or ambulatory care only.

	Hospitalized (n = 116)	Ambulatory (n = 134)	P value	RR
Age (years)	42 (30–52)	36 (27–47)	0.0276*	-
Cp∧ values	18.27 (15.25–21.78)	21.77 (18.53–26.81)	<0.0001*	-
Platelet count×103/µL	131.5 (104.3–176.0)	188.5 (143.8–225.5)	<0.0001*	-
WBC×103/µL	3.1 (2.4–4.4)	4.1 (3.1–6.0)	<0.0001*	-
Lymphocyte count×103/µL	0.5 (0.3–0.6)	0.6 (0.4–0.9)	<0.0001*	-
Neutrophil count×103/µL	2.7 (1.7–3.7)	3.4 (2.5–4.9)	0.0012*	-
IgG seropositivity# (%)	68 (58.6)	53 (39.6)	0.0035	1.5 (1.1–1.9)

Shown are median values with the 25^th^ and 75^th^ percentiles in parentheses.

Chi-square test with Yates' continuity correction was used except where indicated.

*Mann Whitney U test.

∧Cp indicates crossover point on the real-time RT-PCR, which is a semi-quantitative indicator of viremia levels. Lower Cp values indicate higher viremia levels and vice versa.

# IgG seropositivity indicates secondary dengue infection.

WBC indicates white blood cell count.

Among the 116 hospitalized cases, records were available for the 110 patients that were admitted in public hospitals. The remaining 6 patients were treated in private hospitals and their records were not available for review. Of the 110, 20 (18.2%) developed an illness consistent with the classification of severe dengue under the WHO 2009 guidelines [11]. The demographics of these 110 cases are shown in Table 6. Of these 20, 11 (55%) had severe plasma leakage in the form of either a pulse pressure difference of less than 20 mmHg, a systolic pressure of less than 90 mmHg, pleural effusion or ascites, five (25%) bled internally requiring transfusion, three (15%) had liver transaminases that were elevated above 1000 IU and one (5%) had a temporally associated seizure without a previous history of epilepsy.

**Table 6:** Demographics of the hospitalized dengue cases with severe or non-severe dengue.

	Severe dengue N = 20	Non-severe dengue N = 90	P value
Median age (25^th^–75^th^ percentiles)	39.0 (30.3–52.8)	42.0 (30.0–52.0)	NS*
Males (%)	12 (60.0)	57 (63.3%)	NS
Ethnicity			
Chinese (%)	15 (71.2)	68 (59.0)	NS
Malays (%)	2 (7.2)	10 (16.1)	
Indians (%)	2 (13.6)	7 (15.3)	
Others (%)	1 (8.0)	5 (9.6)	
Pre-existing co-morbidity (%)	2 (10.0)	21 (23.3)	NS
Median days hospitalized (25^th^–75^th^ percentiles)	4 (4–5)#	3 (2–4)	0.0095*

Chi-square test with Yates' continuity correction was used except where indicated.

*Mann-Whitney U test.

# One patient was admitted for a total of 37 days due to lack of a caregiver at home. Data from this patient was treated as an outlier and removed from analysis.

### Dengue in different age groups

To examine the impact of increasing age on the clinical presentation and outcome of dengue infection, patients were separated into 5 age groups (18–25, 26–35, 36–45, 46–55 and those 56 years of age and above). The frequency of patients with symptoms associated with dengue fever, namely myalgia, arthralgia, retro-orbital pain and mucosal bleeding reduced significantly with increasing age (Table 7). We have previously shown that the WHO 1997 and 2009 classification schemes are highly sensitive although they lacked specificity [35]. Here, our data indicates that with reducing rates of the above symptoms, the sensitivity of the WHO classification schemes in differentiating dengue from OFI decreased with age (Table 8). Collectively, the results indicate that clinical recognition of dengue becomes harder as the age of the patients increase.

**Table 7:** The early age-specific features and clinical outcomes of dengue patients.

	18–25 (n = 49)	26–35 (n = 60)	36–45 (n = 60)	46–55 (n = 43)	56+ (n = 38)	P value
Drowsiness (%)	33 (67.3)	38 (63.3)	31 (51.7)	27 (62.8)	19 (50.0)	NS
Headache (%)	41 (83.7)	50 (83.3)	51 (85.0)	34 (79.1)	24 (63.2)	NS
Myalgia (%)	35 (71.4)	43 (71.7)	50 (83.3)	25 (58.1)	20 (52.6)	0.0128
Arthralgia (%)	32 (65.3)	44 (73.3)	39 (65.0)	20 (46.5)	17 (44.7)	0.0002
Loss of appetite (%)	42 (85.7)	48 (80.0)	50 (83.3)	37 (86.0)	26 (68.4)	NS
Abdominal pain (%)	7 (14.2)	7 (11.7)	8 (13.3)	5 (11.6)	2 (5.2)	NS
Diarrhea (%)	9 (18.4)	5 (8.3)	13 (21.7)	5 (11.6)	5 (13.2)	NS
Nausea (%)	24 (48.9)	30 (50.0)	33 (55.0)	22 (51.1)	16 (42.1)	NS
Vomiting (%)	5 (10.2)	11 (18.3)	9 (15.0)	9 (20.9)	7 (18.4)	NS
Altered taste (%)	42 (85.7)	48 (80.0)	51 (85.0)	34 (79.1)	28 (73.6)	NS
Rashes (%)	7 (14.3)	7 (11.7)	2 (3.3)	6 (13.9)	2 (5.3)	NS
Retro-orbital pain (%)	22 (44.9)	23 (38.3)	10 (16.7)	6 (13.9)	4 (10.5)	<0.0001
Bleeding (%)	4 (8.2)	7 (11.7)	1 (1.7)	1 (2.3)	0	0.0292
All co-morbidities (%)	1 (2.0)	6 (10.0)	4 (6.7)	9 (20.9)	21 (55.3)	<0.0001
Hypertension (%)	0	1* (1.7)	1 (1.7)	6∧ (14.0)	17∧ (44.7)	<0.0001
Positive IgG (%)	18 (36.7)	16 (26.7)	31 (51.7)	31 (72.1)	27 (71.1)	<0.0001
Hospitalized (%)	22 (44.9)	20 (33.3)	28 (46.7)	23 (53.5)	23 (60.5)	NS
Median days hospitalized (25th–75th percentiles)	5 (5–6)	6 (4–7)	5 (4–6)	5 (4–6)	5 (5–6)	NS*
Severe dengue (%)	3 (6.1)	5 (8.3)	4 (6.7)	4 (9.3)	4 (10.5)	NS
Severe plasma leakage	1	3	3	2	2	NS
Major bleeding	1	1	0	1	2	NS
Organ impairment	1	1	1	1	0	NS

∧Some of these patients have more than one co-morbidities.

Chi square test with Yates' continuity correction was used except where indicated.

*Kruskal-Wallis test.

NS indicates not statistically significant.

**Table 8:** Age-specific features of probable dengue diagnosis using the WHO 1997 or 2009 classification schemes.

WHO 1997	18–25 (dengue n = 49; OFI n = 553)	26–35 (dengue n = 60; OFI n = 499)	36–45 (dengue n = 60; OFI n = 327)	46–55 (dengue n = 43; OFI n–287)	56+ (dengue n = 38; OFI n = 212)
Sensitivity %	95.9 (86.0–99.5	98.3 (91.1–99.9)	95.0 (86.1–99.0)	95.4 (84.2–99.4)	73.7 (56.9–86.6)
Specificity %	32.0 (28.1–36.1)	29.1 (25.1–33.3)	26.3 (21.6–31.4)	35.2 (29.7–41.0)	44.8 (38.0–51.8)
PPV %	11.1 (8.3–14.5)	14.3 (11.1–18.0)	19.1 (14.8–24.1)	18.1 (13.3–23.7)	19.3 (13.2–26.7)
NPV %	98.9 (96.0–99.9)	99.3 (96.3–99.9)	96.6 (90.5–99.3)	98.0 (93.2–99.8)	90.5 (83.2–95.3)

WHO 2009					
Sensitivity %	95.9 (86.0–99.5)	96.7 (88.5–99.6)	96.7 (88.5–99.6)	100 (91.8–100)	81.6 (65.7–92.3)
Specificity %	23.0 (19.5–26.7)	19.9 (16.5–23.7)	19.0 (14.9–23.6)	22.7 (17.9–27.9)	34.9 (28.5–41.7)
PPV %	9.9 (7.4–13.0)	12.7 (9.8–16.1)	18.0 (13.9–22.6)	16.2 (12.0–21.2)	18.3 (12.8–25.0)
NPV %	98.5 (94.5–99.8)	98.0 (93.0–99.8)	96.7 (89.2–99.6)	100 (94.5–100)	91.4 (83.0–96.5)

PPV and NPV denote positive and negative predictive values, respectively.

Values in parentheses are the 95% confidence intervals.

**Figure 1:**
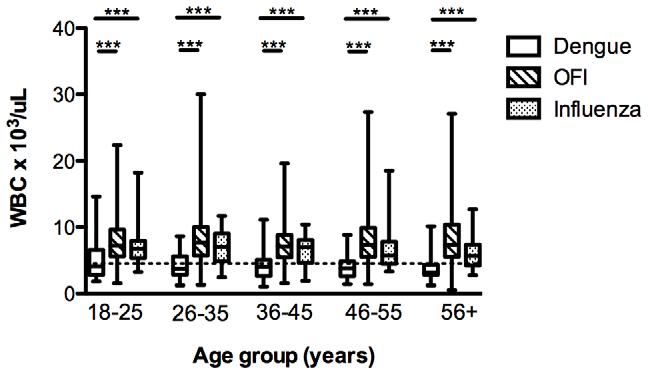
Age-group specific WBC in patients with dengue, OFI and influenza in the first 72 hours of illness. Box and whiskers (maximum and minimum) indicate the mean and spread of WBC in the different groups of patients. Dotted line indicates threshold for leukopenia (WBC<4,500 cells/µL). ***indicates p<0.0001 as determined using the Mann Whitney U test.

We also observed that leukopenia was more marked with increasing age, although this difference was not statistically significant among the dengue patients in different age groups (Figure 1). However, when compared to patients with OFI and influenza in the same age groups, the difference in WBC appeared greater with increasing age (Figure 1). We thus tested if the use of leukopenia alone can differentiate dengue from OFI. Using a receiver operating characteristic (ROC) analysis, the area under the curve (AUC) values increased with age (Table 9). Likewise, the sensitivity of this test increased from 53.1% in the 18–25 year old group to 81.6% in those 56 years old and above. Specificity was over 85% in all age groups (Table 9). Significant differences in the rates of those with leukopenia were also observed across the age groups when comparing dengue and influenza patients (Figure 1). ROC analysis of platelet, neutrophil and lymphocyte counts also showed statistically significant AUC but these were all lower than WBC alone (data not shown).

**Table 9:** Age-specific features of using fever and leukopenia for probable dengue diagnosis.

	18–25 (dengue n = 49; OFI n = 553)	26–35 (dengue n = 60; OFI n = 499)	36–45 (dengue n = 60; OFI n = 327)	46–55 (dengue n = 43; OFI n-287)	56+ (dengue n = 38; OFI n = 212)
Receiver operating characteristic AUC	0.78 (0.71–0.86)	0.87 (0.83–0.91)	0.85 (0.79–0.90)	0.87 (0.81–0.92)	0.91 (0.86–0.96)
Sensitivity %	53.1 (38.3–67.5)	71.7 (58.6–82.6)	63.3 (49.9–75.4)	65.1 (49.1–79.0)	81.6 (65.7–92.3)
Specificity %	89.3 (86.5–91.8)	87.0 (84.3–90.3)	85.1 (80.7–88.7)	88.9 (84.6–92.3)	86.7 (81.6–91.1)
PPV %	30.6 (21.1–41.5)	41.0 (31.5–51.0)	43.7 (33.1–54.7)	46.7 (33.7–60.0)	52.5 (39.1–65.7)
NPV %	95.6 (93.4–97.2)	96.3 (94.1–97.8)	92.7 (89.1–95.4)	94.4 (91.0–96.9)	96.4 (92.6–98.5)

Leukopenia is defined as WBC<4,500 cells/µxL.

AUC indicates area under the curve.

PPV and NPV denote positive and negative predictive values, respectively.

Values in parentheses are the 95% confidence intervals.

## Discussion

In Singapore, DHF was first reported in the 1960s and quickly became a major cause of childhood mortality. With the implementation of vector control leading to reduced *Aedes aegypti* population, the incidence of DHF declined and Singapore experienced a 15-year period of low DF/DHF incidence [15]. However, since the 1990s, dengue has re-emerged as a consequence of a number of different factors [15], [36], [37] and the highest incidence has been observed in adults with relatively few pediatric cases. This setting provides us with an opportunity to examine the clinical features of dengue in adults.

We structured a prospective study enrolling adults presenting with an acute febrile illness of less than 72 hours duration, with follow up over a 3–4 week period to determine the clinical outcome. This enabled us to capture the early features of dengue illness and systematically compare them to other febrile illnesses including influenza, which is another viral infection commonly encountered in the primary healthcare setting. Though Singapore experienced an outbreak of chikungunya from mid-2008 to early 2009 [38], none of our cases tested positive to chikungunya virus.

A limited subset of these patients have been previously analysed and reported elsewhere. These reports have either described the study design along with the preliminary clinical and epidemiological description of the adult dengue cases [30], the development of a algorithms for early dengue diagnosis and triaging for case management [39] or a cross-sectional comparison on the usefulness of NS1 rapid test relative to clinical diagnosis [35], [40]. However, a full analysis of the patients enrolled over a 5-year period has not been previously described.

Our data indicates that clinical features associated with dengue are relatively common during the first 72 hours of illness, which represents the first time patients with acute febrile illness seek medical attention. We have recently shown that the high sensitivity of the WHO dengue classification schemes can be useful in ruling out cases of acute febrile illness from further laboratory investigation for a confirmatory dengue diagnosis [35]. Our findings here indicate that while this is true for most age groups, caution needs to be exercised in older adults as the frequency of symptoms and signs in the WHO classification schemes reduced significantly with increasing age of infection. This would thus render the process of early diagnosis more difficult, as was suggested in a case report [41], thereby reducing the effectiveness of any antiviral intervention [6] when these become available.

The longitudinal study also enabled us to assess the burden dengue imposes on the adult population. The median age of the dengue cases was 39 years old, indicating that the majority of dengue cases are in the productive working age. Overall, dengue patients were ill for a longer period, had greater rates of hospital admission and, if admitted, were hospitalized for a longer period than OFI or influenza. The risk of hospitalization also appeared to increase with age. Given the trend of increasing age of dengue cases in Singapore [15], the burden dengue poses on society is thus likely to worsen.

These considerations, however, may be confounded by the availability of a national guideline for admitting dengue patients for hospitalized treatment. In contrast, the decision to hospitalize patients with OFI or influenza is based solely on the clinical judgement of the emergency physician. Indeed, it is entirely possible that the present guidelines have resulted in over-hospitalization of dengue cases as out of the 110 hospitalized cases reviewed, only 20 (18%) had severe dengue. Furthermore, pre-existing chronic illness are more common in older adults and this criterion in the guideline could have resulted in the increased hospitalization rates with age. However, our study did also observe an increasing trend of severe dengue with increasing age, suggesting that age alone may have an impact on disease outcome. In a retrospective study of the 1981 DHF outbreak in Cuba, peak mortality rates were observed in children and in adults above 60 years old [42]. The underlying mechanism on how age influences clinical outcome, with or without pre-existing chronic illness, however, cannot be gleaned from this study as there were relatively few severe dengue cases.

To improve clinical suspicion of dengue in older adults who present with acute febrile illness, we suggest the use of a simple WBC. Leukopenia has been previously reported to be associated with dengue infections and its use as a diagnostic tool was proposed before the availability of RT-PCR or NS1 antigen assays [43]. The ROC analyses in the different age groups in this study indicate that the usefulness of leukopenia in aiding an early clinical diagnosis of dengue is not consistent throughout all age groups but instead increases sharply with age. Platelet count is not below the normal limits at this stage of the illness in most patients, although it was significantly lower in patients with dengue compared to OFI or influenza, and the usefulness of thrombocytopenia in triggering a differential diagnosis improves in the later stages of illness. While the underlying explanation for the increasing trend of leukopenia with age is not known, it could be an inexpensive tool to enable clinicians to decide whether to pursue additional laboratory tests for dengue or not. It would also be interesting to explore if fever and leukopenia, plus one or more of the list of symptoms in the WHO 2009 classification, could be used for a clinical diagnosis of probable dengue in this group of adults.

To our knowledge, this is the largest prospective study that examined early clinical diagnosis of dengue in adults in a primary healthcare setting [44]. A limitation of this study, however, is the small number of cases that met the WHO 2009 classification's criteria for severe dengue. We have thus not analysed here how predictive the early clinical or haematological parameters are in determining the development of severe illness, which has been addressed elsewhere [39], [45], [46].

In conclusion, early clinical diagnosis based on previously defined symptoms that are associated with dengue, even in the schematics of both the WHO 1997 and 2009 classifications, is difficult in older adults. The presence of leukopenia in older adults that present with an acute febrile illness should trigger a differential diagnosis of dengue for further laboratory confirmation.
